# Generation of Cross-Specialty Consensus Statements on Soft Tissue Management via a Modified Delphi Method

**DOI:** 10.1007/s00268-022-06627-5

**Published:** 2022-07-13

**Authors:** Damir Matic, Joseph S. Cheng, Olivier Gauthier, Yves Harder, Salvatore C. Lettieri, Sandipan Chatterjee, Maio Chen, David Volgas

**Affiliations:** 1 Division of Plastic and Reconstructive Surgery, Department of Surgery, London Health Science Center, Victoria Hospital, Western University, 800 Commissioners Rd E, London, ON N6A5W9 Canada; 2 Department of Neurosurgery, University of Cincinnati College of Medicine, P.O. Box 670515, Cincinnati, OH 45267-0515 USA; 3 Department of Small Animal Surgery and Anesthesia, Food Science and Engineering, ONIRIS Nantes-Atlantic College of Veterinary Medicine, Site de la Chantrerie, 101 route de Gachet - CS 40706, 44307 Nantes cedex 3, France; 4 Department of Plastic, Reconstructive and Aesthetic Surgery, Ospedale Regionale Di Lugano, Sede Ospedale Italiano, Ente Ospedaliero Cantonale (EOC), Via Capelli, CH-6962 Viganello - Lugano, Switzerland; 5 Faculty of Biomedical Sciences, Università Della Svizzera Italiana (USI), Via Buffi 13, 6900 Lugano, Switzerland; 6 Division of Plastic Surgery, Mayo Clinic, 5777 E. Mayo Blvd, Phoenix, AZ 85054 USA; 7 AO Education Institute, AO Foundation, Stettbachstrasse 6, 8600 Dübendorf, Switzerland; 8 AO Innovation Translation Center, AO Foundation, Clavadelerstrasse 8, 7270 Davos, Switzerland; 9 Department of Orthopaedic Surgery, University of Missouri – Columbia, 3800 S National Ave Suite 600, Springfield, MO 65807 USA

## Abstract

**Background:**

Soft tissue management (STM) training programs for surgeons are largely tradition based, and substantial differences exist among different surgical specialties. The lack of comprehensive and systematic clinical evidence on how surgical techniques and implants affect soft tissue healing makes it difficult to develop evidence-based curricula. As a curriculum development group (CDG), we set out to find common grounds in the form of a set of consensus statements to serve as the basis for surgical soft tissue education.

**Methods:**

Following a backward planning process and Kern’s six-step approach, the group selected 13 topics to build a cross-specialty STM curriculum. A set of statements based on the curriculum topics were generated by the CDG through discussions and a literature review of three topics. A modified Delphi process including one round of pilot voting through a face-to-face CDG meeting and two rounds of web-based survey involving 22 panelists were utilized for the generation of consensus statements.

**Results:**

Seventy-one statements were evaluated, and 56 statements reached the 80% consensus for “can be taught as is.”

**Conclusions:**

Using a modified Delphi method, a set of cross-specialty consensus statements on soft tissue management were generated. These consensus statements can be used as a foundation for multi-specialty surgical education. Similar methods that combine expert experience and clinical evidence can be used to develop specialty-specific consensus on soft tissue handling.

## Introduction

During the past 60 years, surgeons and researchers have described two fundamental types of bone healing, primary and secondary. Implants and techniques were developed to reflect the understanding of these basic principles. As the concepts progressed, research was conducted to determine how surgical techniques and implant design might enhance the treatment of fractures according to a better understanding of the biomechanical and biophysiologic healing of fractures. Minimally invasive surgical techniques and other innovations directed an armamentarium for treating various types of fractures with improved outcomes by decreasing the soft tissue damage from open exposures. In parallel, principles-based curricula were developed, and together they led to success in consistent treatment of fractures across disciplines. Today spine, craniomaxillofacial (CMF), orthopedic, and veterinary surgeons approach fracture care in similar ways with a common understanding of the principles of bone healing.

In contrast, in soft tissue management (STM), anecdotal experience (generally accepted as true but may vary among specialties) has been passed on through apprenticeship teaching along with findings based on published evidence and has thus been secondary to the primary focus of bone treatment. After surgical site infection (SSI) was recognized as a major (and sometimes avoidable) problem that can lead to patient morbidity and mortality and economic burden, many reports were published on the treatment of SSI and STM education [1, 2]. Nevertheless, with the lack of clinical data and the great diversity of soft tissue injuries, tradition-based STM training programs prevailed. Thus, substantial differences still exist in soft tissue handling among surgical specialties and became a major challenge in creating unified principles—as we have experienced as a curriculum development group (CDG) representing various surgical specialties.

To overcome this difficulty for the future, we sought to find a common ground in a set of consensus statements that could serve as the basis for a cross-specialty curriculum, designed through a backward planning process [3]. In consultation with a panel of experts, the CDG generated a panel of statements based on the curriculum topics. The statements were then processed using the basic tenet of the Delphi method as has been frequently done in clinical setting when little or no definitive evidence exists, but expert opinions are important [4–6].

This article describes the first steps in building a cross-specialty consensus in essential areas of STM. The resulting statements can be used as a basis for developing STM curricula across disciplines and to identify potential research needs in STM.

## Materials and methods

### Organization

The CDG was responsible for selecting the STM topics and formulating the initial statements for a cross-specialty curriculum. The 6 CDG members were recommended by their colleagues as surgeons with expertise on soft tissue handling and ample experience in teaching the topics. The group included 3 plastic surgeons, 1 spine surgeon, 1 trauma surgeon, and 1 veterinary surgeon.

A panel of 28 international experts (i.e., the panelists, including the 6 CDG members) were invited to comment on the statements and participate in a survey. The panelists were selected for their prior experience in surgical education with emphasis on STM and were encouraged to provide literature evidence that supported or contradicted the statements. Among the panelists, 7 (25.0%) were plastic surgeons (including CMF surgeons), 4 (14.3%) were spine surgeons, 14 (50.0%) were trauma surgeons, and 3 (10.7%) were veterinary surgeons. An education specialist and a medical writer familiar with the Delphi method were included as facilitators.

### Generation of the STM curriculum and consensus statements

The CDG met in person in 2018 and 2019 to define a cross-specialty STM curriculum using a backward planning process and the 6-step approach to curriculum design proposed by Kern [3, 7]. Subsequently, a first draft of statements, which had been sent to the panelists for their feedback and contribution of supporting evidence, was generated by the CDG to go through the following modified 4-stage Delphi process: (1) pilot voting: paper-based pilot voting by the CDG in-person to fine-tune the statements and to select topics for which clinical evidence was likely available for a literature review; (2) round 1 of web-based voting by the panelists; (3) in-person meeting of the CDG to examine evidence resulted from the literature review and formulate statements for round 2 of web-based voting; and (4) round 2 of web-based voting by the panelists.

All voting rounds were anonymous, so panelists could vote without the influence of others. (In theory, the 2 facilitators could identify how the panelists voted.) For each stage, the threshold was set to 80% agreement for the statement to pass as consensus.

### Pilot voting

Before the CDG members met to participate in pilot voting on the initial statements, the members had received and read feedback from the panelists. The 2 facilitators conducted the meeting and recorded the voting results. The voting categories, based on the degree of confidence of a statement being true, were (1) statement can be taught as is, (2) statement can be taught with caution, (3) statement is controversial, and (4) statement should be eliminated.

If a statement received a combined vote of 80% or more for “statement can be taught as is” and “statement can be taught with caution,” the statement was retained and discussed further. The medical accuracy of the statements was verified by the CDG members, and the clarity of the language adjusted. The most controversial topics that may benefit from clinical evidence were selected for literature review. The wording of the statements was finalized by a language/education specialist before the CDG gave final approval.

### Voting, rounds 1 and 2

For round 1 voting, the survey was set up in a web-based electronic data capture system [8] and sent to the panelists. Survey results were exported for descriptive statistics analyses using a user-written SAS program (SAS Institute Inc). The possible categories of votes were the same as those in the pilot voting except for the additional free-text field where panelists could provide comments. Statements that received a minimum of 80% vote for “statement can be taught as is” became consensus statements. Statements that did not reach consensus were discussed at an in-person CDG meeting and were revised (with input from the panelists’ written comments) for round 2 voting or eliminated. Additional statements were formulated from the results of the literature review.

Round 2 voting was conducted similarly except that several new, clinical evidence-based statements were added and key publications from the literature review were sent to the panelists.

### Literature review

A non-systematic literature search was performed using the PubMed database for topics that were selected by the CDG during the pilot voting. The focus of the search was good-quality meta-analyses and recent review articles, but other types of articles were also reviewed.

## Results

### Topics and statements

Thirteen topics from the STM curriculum were selected, and 92 initial statements were formulated by the CDG. The topics were (1) wound types and clinical aspects of wound healing; (2) skin preparation and patient positioning; (3) suture materials (including barbed sutures); (4) methods of hemostasis; (5) surgical incision and exposure; (6) infection in surgical and traumatic wounds; (7) mobilization strategy; (8) penetrating wounds; (9) modifiable factors to optimize wound healing; (10) management of subacute and chronic wounds; (11) postoperative scar management; (12) treatment of soft tissue deformities and symptomatic scars; and (13) skin grafting and flaps.

After review of feedback from the panelists and the pilot voting, statements that reached the 80% approval cutoff were revised by clarifying the wording, splitting into multiple statements, or consolidating into 1 statement. After other statements and redundancies were eliminated (Table 1), 64 statements remained for round 1 voting.

**Table 1:** Statements eliminated after pilot testing

Topic	Statement	Reason for elimination
1 Wound types and clinical aspects of wound healing	1.4 Wound-healing is a complex interplay of cytokines, growth factors that balance well production and degradation	Vague, unclear statement
	1.6 In terms of risk of dehiscence, wound healing of 2 weeks is sufficient prior to starting radiation or chemotherapy after surgery	• The timing is controversial • Oversimplification of statements; dependent on factors such as the location, comorbidities, and types of chemotherapy
2 Skin preparation and patient positioning (Many of the skin disinfection statements were consolidated into 2 statement: follow manufacturer’s direction and allow them to dry. The statement didn’t pass in the end)	2.3 Chlorhexidine, octenidine dihydrochloride and iodine-povidone-iodine have a similar effect of skin decontamination	• Controversial
	2.4 Avoid alcohol-based disinfectant in the face (oral mucosa, conjunctiva)	• Tips and tricks, no evidence
	2.5 Do not combine chlorhexidine and iodine-based disinfectant (purple discoloration)	• Tips and tricks, no evidence
	2.6 Disinfect surgical field at least 2 × 1 min	• Tips and tricks, no evidence
	2.11 For prone positioning, use of chest and abdominal rolls or frames to lower venous pressure and bleeding, with less need for hemostatic techniques which may hinder wound healing	• Statement is too simplistic
	2.12 Three consecutive applications of the antiseptic solution is an appropriate number before draping to ensure surgical site antiseptic preparation	• Tips and tricks, no evidence
	2.14 What kind of draping is to be recommended: single-, double-layered, textile, single use, adhesive, incision adhesive drape, adhesive antiseptic impregnated drape?	• Tips and tricks, no evidence
3 Suture materials	3.7 Sutures are significantly better than tissue adhesives for minimizing dehiscence	Statement revised substantially into “Cyanoacrylate tissue adhesives are used to maintain superficial skin approximation only.”
4 Methods of hemostasis	4.1 Suture ligature is the best way to control large vessel bleeding	
	4.2 Electrocautery can be used to stop all forms of bleeding	Too general, too sweeping
	4.6 Skin adhesives should not be used without an appropriate approximation of the underlying muscular, sub cutaneous and dermal tissues	
	4.7 Skin adhesives are only indicated to close the epidermal surface of low-tension skin lacerations and surgical incisions	
	4.8 Advanced-energy tissue/vessel sealing and dissection devices (thermal fusion, ultrasonic instruments) provide medical benefits for the patients and economic benefits for health economy once they are properly used. This proper use needs education and training	Not applicable for Trauma
	4.9 Advanced-energy tissue and vessel sealing devices (thermal fusion, ultrasonic device) may be appropriate to control or coagulate single large vessel bleeding (up to 5, 6, 7 mm in diameter ?)	Not applicable for Trauma
	4.11 Electrocautery in cutting mode and at lower setting as possible can be used for skin incisions	Lack of evidence
5 Surgical incision and exposure	5.3 Poor application of retractors or the use of the wrong size of tissue retractor results in skin edge necrosis, blood supply compromise and can tear skin	
	5.9 The half-buried vertical mattress has the least adverse effect on skin blood flow	Not always true; depends on the comparators
8 Penetrating wounds	8.1 Hydrosurgical debridement is an effective method to debride contaminated tissues	Disputed topic
	8.3 What is the role of drains in internal degloving injuries?	More evidence needed
9 Modifiable factors to optimize wound healing	9.5 The use of honey on partial thickness and contaminated wounds reduces infection rates	More evidence needed
11 Postoperative scar management	11.3 Incisions need to be kept clean	Too simplistic

Statements related to the topics of operating room behavior, barbed sutures, and negative pressure wound therapy are not listed here

The pilot voting identified 3 topics that were considered controversial but likely to have evidence in the literature: (1) operating room (OR) behavior, (2) barbed sutures (application and clinical results), and (3) application of negative pressure wound therapy (NPWT). Most statements under these topics were temporarily removed from the survey and were later reformulated for round 2 voting according to the results of the literature review. Examples of search terms for OR behavior were “operating room,” “door opening,” “surgical site infection,” “attire,” and “jewelry.” Examples for barbed sutures were “barbed sutures,” “wound closure,” “wound dehiscence,” “comparing,” and “outcome.” Examples for NPWT were “negative pressure wound therapy,” “wound contracture,” “wound healing,” “benefit,” “tissue granulation,” and “contraindicat*.”

### Literature review

For each of the 3 topics, 25–30 full-text publications were evaluated. The results were collated and discussed at a CDG meeting. The collated results and a set of literature on OR behavior [9–11], barbed sutures [12–15], and NPWT [16–18] were sent to the panelists before round 2 voting.

### Survey results

**Fig. 1:**
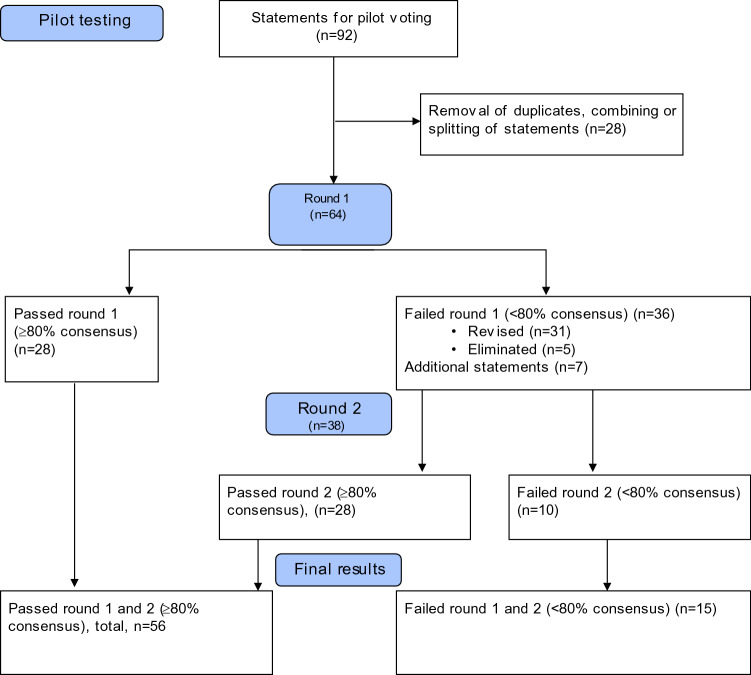
Flow chart showing status of statements

The number of statements at each voting stage is summarized in Fig. 1. Of the 28 panelists, 22 (78.6%) answered the round 1 survey. These 22 participants represented 4 surgical disciplines: plastic surgery (including CMF surgery) (*n* = 6; 27.3%); spine surgery (*n* = 3; 13.6%); trauma surgery (*n* = 10; 45.5%); and veterinary surgery (*n* = 3; 13.6%). Geographically, they were from North America (8), Central and South America (3), Europe (5), Asia (4), and Middle East (2). Of the 64 statements, 28 passed the 80% cutoff and could be taught without revision. Among the statements that did not reach the cutoff, 31 were revised for the round 2 survey and 5 were eliminated without further voting. The CDG formulated 7 new statements from the evidence stemming from the literature review. In total, 38 statements for round 2 voting were sent to the 22 panelists who had participated in the first round.

The return rate for round 2 voting was 100%. Of the 38 statements included in the round 2 survey, 28 passed the 80% cutoff, so they could be taught without revision, and 10 statements did not pass the 80% cutoff. In summary, 56 statements (28 from round 1 and 28 from round 2) could be taught without revision. Table 2 summarizes these 56 consensus statements (in their final wording), and Table 3 summarizes the statements that did not make the cutoff along with selected comments from the panelists. A panelist may have voted for a statement to be taught as is but nevertheless wrote a comment, so these comments do not necessarily reflect how a panelist voted. Nevertheless, these comments supplement the statements with points to consider for teaching and may provide clues as to why a statement did not reach consensus.

**Table 2:** Summary of survey results for all statements

Statement	Results^a^	Respondents, %			
		Statement can be taught as is	Statement can be taught with caution	Statement is controversial	Statement should be eliminated
*Wound types and clinical aspects of wound healing*					
1.1 The degree of wound contamination is one factor that has a direct effect on the surgical management of the soft tissues	Passed, 2nd round	91	5	5	0
1.2 Local and systemic factors can inhibit wound healing and scar maturation	Passed, 1st round	90	10	0	0
1.3 A previously infected wound that has been turned into a clean granulating wound can be closed using any appropriate method	No consensus	73	27	0	0
*Skin preparation and patient positioning*					
2.1 The presence of hair at the surgical site does not increase the risk of infection. When necessary, hair should be removed by clipping rather than shaving	Passed, 2nd round	86	9	5	0
2.2 Hair clipping as opposed to shaving is the preferred method and does not increase wound complication rates	Passed, 1st round	81	14	5	0
2.3 Skin preparation solutions are effective as long as the manufacturers’ instructions are followed	No consensus	77	18	5	0
2.4 Prep and drape the surgical field generously to allow extension of the incision if needed	Passed, 1st round	86	14	0	0
2.5 Regardless of the type of skin preparation used, the solution should be allowed to dry prior to draping the surgical field for maximal effectiveness and other considerations such as sterility and fire prevention	Passed, 2nd round	86	14	0	0
2.6 Adequate padding of bony prominences during patient positioning reduces pressure-related complications	Passed, 1st round	90	10	0	0
2.7 Position all patient extremities without tension in order to reduce the risk of neural plexus injury	Passed, 1st round	86	10	5	0
*Suture materials*					
3.1 Resorbable braided sutures should be avoided in contaminated wounds	Eliminated	57	24	19	0
3.2 Non-resorbable monofilament sutures produce the least inflammatory response when used for skin closure	Passed, 2nd round	91	9	0	0
3,3 The thinnest suture diameter that does not break during wound approximation is the most appropriate for wound closure	Passed, 2nd round	82	14	5	0
3.4 Multiple, evenly spaced, interrupted sutures reduce tension at the wound edge during closure more effectively than a smaller number of larger sutures	Passed, 1st round	81	5	10	5
3.5 Cyanoacrylate tissue adhesives are used to maintain superficial skin approximation only	Passed, 2nd round	91	5	5	0
*Methods of hemostasis*					
4.1 Surgical wound closure (including the skin) should be achieved in a layered closure whenever possible	Passed, 2nd round	91	5	5	0
4.2 Washing a surgical wound with soap and water 48 h after primary wound closure does not lead to increased infection rates	No consensus	73	23	5	0
4.3 Low-pressure expandable hemostats, such as flowable gelatins, should be used with thrombin for optimal hemostasis	Eliminated	19	38	29	14
4.4 Energy devices generate heat within tissues that is proportional to time and intensity of application. Excessive use will generate collateral tissue damage, increasing the inflammatory response	Passed, 1st round	90	0	0	10
*Surgical incision and exposure*					
5.1 Incisions along relaxed skin tension lines result in the best possible scar after healing	Passed, 1st round	81	19	0	0
5.2 Extending open traumatic wounds/lacerations via acute-angled incisions should be avoided where possible to reduce the risk of compromising skin edge perfusion	Passed, 2nd round	82	18	0	0
5.3 Undermining skin flaps in which the overlying skin is injured (partial thickness loss) increases the risk of full thickness skin necrosis	Passed, 1st round	86	5	10	0
5.4 Horizontal mattress sutures carry a higher risk of skin edge necrosis	No consensus	55	41	5	0
5.5 Skin edge bleeding can be better controlled by using the continuous running suture techniques than the interrupted methods	No consensus	59	23	15	5
5.6 Layered soft-tissue closure with closure of dead space reduces the risk of hematoma formation and wound healing complications	Passed, 1st round	95	0	0	5
5.7 Meticulous hemostasis and dead space closure are more effective wound management techniques than the insertion of drains in reducing complications	Passed, 2nd round	86	14	0	0
5.8 Tissue dissection using a properly powered electrocautery (as opposed to a scalpel) can be more efficient and result in less blood loss	No consensus	50	45	5	0
*Infection in surgical and traumatic wounds*					
6.1 Surgical site infections (SSIs) are the commonest postoperative complications and are responsible for the highest cost of treatment	Passed, 2nd round	91	5	5	0
6.2 The commonest source of SSIs is the patient's skin flora	Passed, 2nd round	86	9	5	0
6.3 The commonest time of diagnosis of an SSI is approximately one week (7–10 days) postoperatively	No consensus	68	23	9	0
6.4 Washing the skin preoperatively with a cleansing solution, such as soap, reduces bacterial count	Passed, 2nd round	86	14	0	0
6.5 Failure to close a dead space during wound closure increases the risk of wound complications	Passed, 2nd round	91	9	0	0
*Mobilization strategy*					
7.1 One cause of prolonged edema in the setting of trauma is impaired lymphatic drainage	Passed, 1st round	81	19	0	0
7.2 The arteriovenous impulse device is effective for reducing postinjury, preoperative edema	Eliminated	48	24	14	14
7.3 Early range of motion of joints after surgery reduces postoperative edema	Passed, 2nd round	86	14	0	0
*Modifiable factors to optimize wound healing*					
8.1 Patient nutrition can affect the risk of wound complications following surgery	Passed, 1st round	100	0	0	0
8.2 Poor perioperative glucose control in diabetic patients increases the risk of postoperative wound complications	Passed, 1st round	95	0	5	0
8.3 Smoking increases the risk of wound complications following surgery	Passed, 1st round	100	0	0	0
8.4 Cessation of smoking approximately 3 weeks before and after surgery reduces the negative effects of smoking on wound healing	Passed, 2nd round	86	14	0	0
8.5 Inadequate debridement of wounds increases wound healing complications	Passed, 1st round	100	0	0	0
8.6 Foreign body retention in wounds increases wound healing complications	Passed, 1st round	86	14	0	0
8.7 Postoperative dressings can be removed at 48 h after surgery because a normal healing surgical wound will have sealed through re-epithelialization	No consensus	59	36	5	0
8.8 Platelet-rich plasma has been shown to have minimal benefit in reducing wound healing complications in most cases	Passed, 2nd round	82	9	9	0
8.9 Hyperbaric oxygen has minimal benefit in reducing wound healing complications in most wounds	No consensus	73	14	9	5
8.10 Nitropaste has been shown to have minimal benefit in reducing wound healing complications in most cases	Passed, 2nd round	86	9	5	0
*Management of subacute and chronic wounds*					
9.1 Bacterial load reduction aids in wound healing	Passed, 1st round	95	5	0	0
9.2 Residual necrotic tissue impedes wound healing	Passed, 1st round	90	10	0	0
9.3 Early, radical debridement of non-viable tissue compared to the "wait and see" approach reduces wound complications, promoting wound healing	Passed, 1st round	90	10	0	0
9.4 Wounds heal best in a moist, clean, warm environment	Passed, 1st round	95	5	0	0
*Postoperative scar management*					
10.1 Postoperative scarring is worse when the wound is closed under tension	Passed, 1st round	95	5	0	0
10.2 Postoperative scarring is worse in wounds that have healed after postoperative infection	Passed, 1st round	86	10	5	0
10.3 Postoperative scarring is worse in wounds that have healed by secondary intention	Passed, 1st round	86	14	0	0
10.4 Sunscreen is helpful in minimizing pigmentation changes in maturing scars	Passed, 1st round	81	14	5	0
10.5 Intralesional steroid injections reduce scar tissue and may improve the appearance of hypertrophic and keloid scars	Passed, 2nd round	95	5	0	0
*Treatment of soft-tissue deformities and symptomatic scars*					
11.1 Topical scar treatments, such as silicon sheeting, may help minimize and reduce scarring	Passed, 2nd round	86	9	5	0
11.2 Scars with functional compromise need to be corrected prior to full maturation	Eliminated	67	24	10	0
11.3 Scar revisions are best performed when the scar has completely matured	Passed, 2nd round	82	18	0	0
11.4 Topical application of silicon sheeting has been shown to improve the appearance of hypertrophic scars	Eliminated	67	19	14	0
*Skin grafting and flaps*					
12.1 Skin grafts can provide efficient coverage of wounds	Passed, 2nd round	81	19	0	0
12.2 Skin grafting is contraindicated for poorly vascularized wound beds such as exposed bone	Passed, 1st round	90	5	5	0
12.3 Skin grafting is contraindicated for covering vital structures such as exposed vessels	Passed, 1st round	90	10	0	0
12.4 Skin grafting is contraindicated for covering exposed hardware	Passed, 1st round	100	0	0	0
12.5 Partial thickness grafts contract more than full thickness grafts	Passed, 1st round	86	14	0	0
12.6 Skin flaps provide better long-term stability for wound coverage than skin grafts	Passed, 2nd round	95	5	0	0
*Statements based on literature reviews, voted only in the second round*					
*Operation room behavior*					
13.1 Reasonable evidence exists that the number of OR door openings during a procedure is associated with increased SSI rates	Passed, 2nd round	95	5	0	0
13.2 Variables such as gloves, masks, surgical hats, and wearing jewelry in the operating room have not been shown to influence SSI rates	No consensus	64	32	5	0
*Negative pressure wound therapy*					
14.1 Negative pressure wound therapy (NPWT) may function through multiple mechanisms, such as tissue perfusion changes, exudate control, stimulation of granulation tissue formation, and wound size reduction	Passed, 2nd round	100	0	0	0
14.2 Existing evidence suggests a potential association of NPWT with reduced wound healing complications	Passed, 2nd round	91	5	5	0
14.3 NPWT may lead to increased adverse effects in certain conditions such as wounds at high risk for bleeding, exposed viscera, vessels and vascular anastomoses, necrotic wound beds, untreated osteomyelitis, and malignancy	Passed, 2nd round	95	5	0	0
*Barbed sutures*					
15.1 Although evidence exists that using barbed sutures may save operative time, the evidence is inconsistent and dependent on factors such as type of surgery, type of wound, layer of closure, surgeon experience, and patient factors	Passed, 2nd round	95	5	0	0
15.2 Currently, there is insufficient evidence to answer whether barbed sutures are associated with more or less wound-related complications compared to traditional sutures	Passed, 2nd round	95	5	0	0

^a^Of the 71 statements, 28 passed in round 1 and 28 passed in round 2. *Eliminated* indicates that the statement was eliminated after round 1 (*n* = 5). *No consensus* indicates that consensus was not reached after 2 rounds of voting (*n* = 10)

**Table 3:** Failed statements and selected comments

Statements (% approval^a^)	Comments^b^ (surgical specialty)
Round 1	
1.1 The surgical management of soft tissues is dictated by the degree of wound contamination (57%)	• … also soft tissue injury. They are often, but not always related (Trauma) • …other factors: implant, radiation, the need for future surgery…(CMF, VET)
1.3 A previously infected wound that has been turned into a clean granulating wound can be closed (48%)	• What is “a clean wound”? Define closing. (Trauma) • It depends on other factors: underlying hardware, closure type (Trauma, CMF) • Granulation tissue colonized with bacteria will need excision prior to closing or grafting (CMF)
2.1 The presence of hair at the surgical site does not increase the risk of infection (62%)	• Strong Evidence for “hair at the surgical site should be left in place (Spine) • Does not apply to vets: hair removal is a must (VET)
2.3 Commercially available skin preparation solutions are effective provided the manufacturers' guidelines are followed correctly (43%)	• Differences between various prep solutions should be pointed out (Trauma) • Moderate evidence shows that a safe, effective health care organization-approved antiseptic should be selected for individual patients (Spine)
2.5 Regardless of the disinfectant used, this should be allowed to dry prior to draping the surgical field (67%)	• I believe that alcohol kills on contact (Trauma) • Prior to incision? (Spine) • For sterility or fire prevention? (Trauma)
3.1 Resorbable braided sutures should be avoided in contaminated wounds^c^ (57%)	• Proper debridement is key; sutures play a minor role (CMF) • “Braided suture causes infection” is passe…. (CMF) • … One cannot avoid them completely… (Trauma)
3.2 Non-resorbable monofilament sutures should be used for skin closure as they produce the lowest amount of inflammatory response (67%)	• Depends on wound (CMF) • Suture type is more important than the material (CMF) • For continuous intradermal sutures, resorbable material can also be used (Trauma)
3.3 The thinnest suture diameter that does not break during wound approximation is the most appropriate for wound closure and produces the lowest amount of inflammatory response (67%)	• Does not break and does not cut through tissues (Trauma) • Smallest needle size should also be chosen to minimize tissue damages (VET)
3.5 Cyanoacrylate tissue adhesives allow approximation that is limited to the epidermis (76%)	• Often this device is used as a crutch (CMF) • Epidermis and superficial dermis (Trauma)
4.1 Surgical wound closure should be a layered closure which includes the intradermal layer (76%)	• What is the intradermal layer? (Trauma) • It depends on the thickness of the tissue. Eyelid skin is too thin for layered closure… (CMF)
4.2 Washing a wound with soap and water 24–48 h after wound closure does not lead to increased infection rates (33%)	• This may not be true in all settings (Trauma) • Depending on the wounds and patient condition (CMF) • Habits could be different from hospitals, countries, surgeons (VET)
4.3 Low pressure expandable hemostats such as flowable gelatins should be used with thrombin for optimal hemostasis^c^ (19%)	• Flowable gelatins left in place can cause swelling of the tissue by 20% (CMF) • Statement needs to be rephrased and better describe the exact circumstances… (Trauma) • This technique is not currently used by vets (VET)
5.2 In order to extend an open traumatic wound/laceration to avoid compromising the skin edges, where possible, make a right-angled incision (38%)	• Avoid acute angles—somewhat acute is also appropriate (Trauma) • Depending on other factors such as the quality of the skin (CMF, Spine, Trauma) • It depends on multiple factors (Spine) • Revise wording (VET)
5.4 Horizontal mattress sutures carry the highest risk of skin edge necrosis (43%)	• … excessive tension is the ultimate evil (Trauma) • Technique and placement of the sutures influence this much more (CMF) • …a very sweeping statement. (Spine, Trauma) • depending on the technique used (VET)
5.5 Running sutures control skin edge bleeding better than simple interrupted sutures (43%)	• Meticulous haemostasis should be emphasized instead (CMF)
5.7 Postoperative insertion of wound drains does not prevent complications such as hematomas, seromas, and infection (62%)	• … not in every single case… (Trauma) • …misleading… This is a much more complicated scenario than a simple sentence can explain. (CMF)
5.8 Tissue dissection using electrocautery as compared to scalpel can be quicker and result in less blood loss (33%)	• …(it) increases thermal necrosis (Trauma, Spine) • Applied with caution in traumatized overlying skin (Spine, CMF) • The consequences on tissue healing should be mentioned (VET)
6.1 Surgical site infections (SSIs) are the commonest complications that occur postoperatively and are responsible for the highest cost of treatment compared with other postoperative complications (67%)	• Cost factors should not be included (VET)
6.2 The commonest source of SSIs is the patient's own skin flora (71%)	• Contaminations from the injuries are equally to blame (CMF)
6.3 The commonest time of onset of an SSI is approximately one week postoperatively (67%)	• 7–10 days (Trauma) • Define “onset” (CMF)
6.4 Washing the skin with soap and water reduces bacterial counts both pre- and postoperatively (76%)	• To my knowledge, this statement is not supported by any scientific data–true for preop but not postop (Trauma) • Any form of disinfecting solution? (CMF)
6.5 Failure to close a dead space during wound closure increases the risk of postoperative infection (76%)	(none)
7.2 The arteriovenous impulse device is effective for reducing postinjury, preoperative edema^c^ (48%)	• Arteriovenous impulse device is basically use for preventing DVT (Spine) • This device is not used in veterinary orthopedics (VET)
7.3 Early ambulation after surgery reduces the risk of postoperative edema (62%)	• In the upright position gravity leads to an increase of the edema. In critical wounds ambulation is thus not to be recommended. In some instances active motion may even lead to shearing forces in the tissue and thus impair wound healing (e.g., Tibialis anterior in lower leg)…. (Trauma) •…it is one of the factors that may reduce postoperative edema, compression is another, … (Trauma)
8.4 Cessation of smoking 3–4 weeks before surgery and 2–3 weeks postoperatively reverses the negative effects of smoking on wound healing (62%)	• Don’t think there is supportive evidence for this (Trauma) • Irrelevant for vets (VET) • Some say 6 weeks preop (Trauma)
8.7 Postoperative dressings should be removed by 48 h after surgery because a normal healing surgical wound will have sealed through re-epithelialization (57%)	• Statement too broad; it depends on many factors… (Trauma, CMF) • Injudicious manipulation of the wound will cause more injuries and pain (CMF, Trauma) • Statement is based on very low quality evidence from three small randomized controlled trials (Spine) • Should be, or can be? (VET)
8.8 Platelet-rich plasma (PRP) has little or no benefit in reducing wound healing complications in most wounds (57%)	• Disagree. Evidence exists that PRP was safe and cost effective for treating cutaneous wound healing (Ref: PRP: new insights for cutaneous healing) (Trauma) • Many conflicting reports exist that on the potential clinical efficacy of PRP (Spine)
8.9 Hyperbaric oxygen has little or no benefit in reducing wound healing complications in most wounds (62%)	• Conflicting reports, little evidence, for selected situation/wounds only (CMF, Spine, Trauma)
8.10 Nitropaste has little or no benefit in reducing wound healing complications in most wounds (57%)	• Little evidence and maybe true for some highly selected wounds such as diabetic foot ulcers (Trauma)
10.5 Chemotherapy with intralesional steroids improves the appearance of hypertrophic and keloid scars (71%)	• Not always (Spine) • Delete the word “chemotherapy”—it’s not used to improve scars (Trauma)
11.1 Topical scar treatments may help minimize scarring (67%)	• I do not see clear recommendation in this wording (VET)
11.2 Scars with functional compromise need to be corrected prior to full maturation^c^ (67%)	(none)
11.3 Scar revision for aesthetic or psychosocial reasons are best performed when the scar is fully mature (71%)	• Irrelevant for vets (VET) • Not always true (CMF, Trauma)
11.4 Topical application of silicon sheets has been shown to improve the appearance of hypertrophic scars^c^ (67%)	• Not sure there is evidence (Spine)
12.1 Skin grafts can provide efficient coverage of wounds (76%)	• Not always true (Trauma)
12.6 Flaps provide better stability for wound coverage than skin grafting (71%)	• It depends on the situation, size, location, and if stability is a concern (Trauma, CMF)
Round 2	
1.3 A previously infected wound that has been turned into a clean granulating wound can be closed using any appropriate method (73%)	• It depends on degree of granulation, bone exposure, etc. (CMF) • …accurate if we have controlled for contamination causes (CMF) • What's "any appropriate method"? (Spine, Trauma)
2.3 Skin preparation solutions are effective as long as the manufacturers' instructions are followed (77%)	• Not all skin preparation solutions are equally effective (Trauma) • Most hospitals or local authorities have guidelines for the use of these reagents (CMF, Trauma)
4.2 Washing a surgical wound with soap and water 48 h after primary wound closure does not lead to increased infection rates (73%)	• Or it may be even better. A clean wound is better that one with blood on it. (CMF) • It depends on the type of surgical wound… (CMF) • No scrubbing of the skin surface (CMF) • This will be a hard sell for those of us who grew up waiting 10–14 days (Trauma)
5.4 Horizontal mattress sutures carry a higher risk of skin edge necrosis (55%)	• Higher than what? (Spine, Trauma, CMF) • Never had this problem (Trauma) • More importantly, wound closure should be tension free (CMF)
5.5 Skin edge bleeding can be better controlled by using the continuous running suture techniques than the interrupted methods 59%	• But the risk of skin edge necrosis will be higher. Being over zealous in both 5.4 and 5.5 and lead to problems, no circulation = no wound healing (Trauma) • It depends on other factors such as the quality of the suture technique and type of knots (Trauma) • Usually appropriate hemostasis before closure followed by appropriate multilayer closure should have skin edge better controlled. by doing a running suture, although it may increase controlling the bleeding, but not sure if this would be the reason to do such technique (CMF)
5.8 Tissue dissection using a properly powered electrocautery (as opposed to a scalpel) can be more efficient and result in less blood loss (50%)	• It's surgeon (i.e., experience) dependent. (Trauma) • … on a low or moderate energy setting or with a focused tip (e.g., Colorado cautery) (CMF) • Statement regarding appropriate use of coag or cut functions should be made either separately or within this statement (VET) • We try to avoid this in certain areas of face and neck where skin is very thin (CMF) • Occasionally, skillful scalpel dissection is more efficient regarding tissue damage (Trauma)
6.3 The commonest time of diagnosis of an SSI is approximately one week (7–10 days) postoperatively (68%)	• This is the earliest time of SSI diagnosis (CMF) • … our institutions and most in US define a SSI as occurring up to 30 days after surgery. (CMF)
8.7 Postoperative dressings can be removed at 48 h after surgery because a normal healing surgical wound will have sealed through re-epithelialization (59%)	• It depends on many factors such as epithelialization and sealing, wound type, patient factors (CMF, Trauma, multiple comments pointing to the same concept)
8.9 Hyperbaric oxygen has minimal benefit in reducing wound healing complications in most wounds (73%)	• Controversial. HBO does help in patients with compromised vascularity, those with peripheral vascular disease or chronic wounds (CMF, multiple similar comments)
13.2 Variables such as gloves, masks, surgical hats, and wearing jewelry in the operating room have not been shown to influence SSI rates (64%)	• There needs to be some inclusion about facial hair. It is currently a requirement in many ORs to have beards covered (CMF) • Not sure about jewelry (CMF) • These are primarily to protect the surgeon (Trauma)

*CMF* craniomaxillofacial; *VET* veterinary

^a^*Approval* indicates that statement could be taught as is

^b^Comments may not reflect how the participants voted. Comments may not always be direct quotations, they may have been edited for brevity

^c^Statement eliminated after round 1

## Discussion

Through a modified Delphi process, the 6-member, cross-specialty CDG identified 56 statements (from 92 preliminary statements) that reached 80% consensus and could be taught without revision.

### Why did statements fail to reach consensus?

To understand why a statement did not reach consensus and could not be taught without revision, the CDG examined the statements along with the panelists’ comments. Some statements had insufficient detail and needed modification (e.g., statement 1.1, Table 3). Some were too general and should not be taught without qualification (e.g., statement 4.2, Table 1). Some lacked sufficient evidence, and the traditional teaching was too disparate among different surgical fields (e.g., statement 2.1 and statement 4.3, Table 3).

### Wording changes and literature support that promoted consensus

An example of a statement that needed revising is statement 8.10 (“Nitropaste has little or no benefit in reducing wound healing complications in most wounds,” Table 3). Although this concept is commonly taught in the care of compromised skin, the statement did not reach consensus in round 1. After the original statement was qualified by the word minimal, it passed with 86% consensus (Table 2). Another example is statement 13.1 (“Reasonable evidence exists that the number of OR door openings during a procedure is associated with increased SSI rates”). This issue has been debated frequently, and it probably would have failed to reach consensus, but with the support of a literature review, it reached consensus on the first try (Table 2). This statement and its supporting evidence could be used to persuade hospital administrations to introduce the concept into the OR policy.

### Consensus statements across specialties

We want to emphasize that the statements were developed to consider practices of different surgical specialties. While some statements may seem basic, it is reassuring that they are agreed by different specialties, as demonstrated by statements 6.1 (“SSIs are the commonest postoperative complications…”) and 6.2 (“The commonest source of SSIs is the patient’s skin flora”) (Table 2). Other statements may have been basic surgical principles based on experience and handed down as dogma; they have nevertheless reached consensus implying that they are broadly accepted by multiple specialties, such as statements 2.6 (“Adequate padding of bony prominences during patient positioning reduces pressure-related complications”) and 8.5 (“Inadequate débridement of wounds increases wound healing complications”) (Table 2).

While most of the consensus statements represent level V evidence, the use of a modified Delphi method on the experienced educators’ opinions increased the validity of the consensus statements. Smith and Pell [19] pointed out in their tongue-in-cheek article, “the basis for parachute use is purely observational, and its apparent efficacy could potentially be explained by a healthy cohort effect”; with the Delphi method, a statement recommending the use of a parachute would clearly receive greater than 80% consensus and pass in the first round. This example illustrates the many barriers to conducting higher level studies in various clinical areas. Formidable obstacles include the diversity of soft tissue injury (e.g., traumatic or intraoperative), the lack of an easy-to-use and reproducible classification system for soft tissue injury, the lack of detailed documentation of surgical techniques and follow-up, and the lack of funding for research of this type. Even when evidence exists, it is not disseminated well across specialties. In addition, we surgeons are sometimes too quick to blame patient factors (e.g., smoking, diabetes, and vascular disease) for complications with healing or infections rather than our own soft tissue handling techniques.

The purpose of this study was not only to validate a few consensus statements but also to point out how our experience in creating a cross-specialty curriculum had prompted us to reexamine how our education in STM had been done. Through the extensive discussion and a literature review, we believe some well-balanced statements have been created, and these shall be suitable for use in future STM education. The generation of these consensus statements illustrates the “one medicine, one health” concept: There are shared principles across specialties and species [20].

The current work has obvious limitations: First, the selection of the experts was not based on their research credentials in STM but on their experience, interest, and prominence in teaching. Second, choosing precise wording for consensus statements among a group of surgeons who do not share a common first language was challenging. This could have been circumvented by supplementing the statements with examples to ensure an accurate conveyance of the meaning.

## Conclusions

Using a modified Delphi method, we have arrived at a set of cross-specialty consensus statements for STM. These statements are applicable across multiple surgical disciplines and can be used as a foundation for evidence-based surgical education.

## References

[1] Metsemakers WJ, Fragomen AT, Moriarty TF (2020). Evidence-based recommendations for local antimicrobial strategies and dead space management in fracture-related infection. J Orthop Trauma.

[2] Metsemakers WJ, Morgenstern M, Senneville E (2020). General treatment principles for fracture-related infection: recommendations from an international expert group. Arch Orthop Trauma Surg.

[3] Shaye DA, Tollefson T, Shah I (2018). Backward planning a craniomaxillofacial trauma curriculum for the surgical workforce in low-resource settings. World J Surg.

[4] Dalkey N (1969). An experimental study of group opinion: the Delphi method. Futures.

[5] Hasson F, Keeney S, McKenna H (2000). Research guidelines for the Delphi survey technique. J Adv Nurs.

[6] Eubank BH, Mohtadi NG, Lafave MR (2016). Using the modified Delphi method to establish clinical consensus for the diagnosis and treatment of patients with rotator cuff pathology. BMC Med Res Methodol.

[7] Thomas PA, Kern DE, Hughes MT, et al Curriculum development for medical education : a six-step approach, p xii, 300 pages 10.1097/ACM.0000000000002580 30681454

[8] Harris PA, Taylor R, Thielke R (2009). Research electronic data capture (REDCap)–a metadata-driven methodology and workflow process for providing translational research informatics support. J Biomed Inform.

[9] Alizo G, Onayemi A, Sciarretta JD (2019). Operating room foot traffic: a risk factor for surgical site infections. Surg Infect (Larchmt).

[10] Bartek M, Verdial F, Dellinger EP (2017). Naked surgeons? The debate about what to wear in the operating room. Clin Infect Dis.

[11] Moalem J, Markel TA, Plagenhoef J, et al (2019) Proceedings and recommendations from the OR attire summit: a collaborative model for guideline development. Bull American College Surg

[12] Chan VWK, Chan PK, Chiu KY (2017). Does barbed suture lower cost and improve outcome in total knee arthroplasty? A randomized controlled trial. J Arthroplast.

[13] Kim KY, Anoushiravani AA, Long WJ (2017). A meta-analysis and systematic review evaluating skin closure after total knee arthroplasty-what is the best method?. J Arthroplast.

[14] Lin Y, Lai S, Huang J (2016). The efficacy and safety of knotless barbed sutures in the surgical field: a systematic review and meta-analysis of randomized controlled trials. Sci Rep.

[15] Lin Y, Liao B, Lai S (2019). The application of barbed suture during the partial nephrectomy may modify perioperative results: a systematic review and meta-analysis. BMC Urol.

[16] Lalezari S, Lee CJ, Borovikova AA (2017). Deconstructing negative pressure wound therapy. Int Wound J.

[17] Li HZ, Xu XH, Wang DW (2019). Negative pressure wound therapy for surgical site infections: a systematic review and meta-analysis of randomized controlled trials. Clin Microbiol Infect.

[18] Webster J, Liu Z, Norman G, et al (2019) Negative pressure wound therapy for surgical wounds healing by primary closure. Cochrane Database Syst Rev 3:CD009261 10.1002/14651858.CD009261.pub4 PMC6434581 30912582

[19] Smith GC, Pell JP (2003). Parachute use to prevent death and major trauma related to gravitational challenge: systematic review of randomised controlled trials. BMJ.

[20] Gyles C (2016). One medicine, one health, one world. Can Vet J.

